# Protease 3C of hepatitis A virus induces vacuolization of lysosomal/endosomal organelles and caspase-independent cell death

**DOI:** 10.1186/s12860-015-0050-z

**Published:** 2015-02-27

**Authors:** Andrey V Shubin, Ilya V Demidyuk, Nataliya A Lunina, Alexey A Komissarov, Marina P Roschina, Olga G Leonova, Sergey V Kostrov

**Affiliations:** Laboratory of Protein Engineering, Institute of Molecular Genetics, Russian Academy of Science, Moscow, 123182 Russia; Engelhardt Institute of Molecular Biology, Russian Academy of Sciences, Moscow, 119992 Russia; National Research Center “Kurchatov Institute”, Moscow, 123182 Russia

**Keywords:** 3C protease, Hepatitis A virus, Cytoplasmic vacuolization, Caspase-independent cell death

## Abstract

**Background:**

3C proteases, the main proteases of picornaviruses, play the key role in viral life cycle by processing polyproteins. In addition, 3C proteases digest certain host cell proteins to suppress antiviral defense, transcription, and translation. The activity of 3C proteases *per se* induces host cell death, which makes them critical factors of viral cytotoxicity. To date, cytotoxic effects have been studied for several 3C proteases, all of which induce apoptosis. This study for the first time describes the cytotoxic effect of 3C protease of human hepatitis A virus (3Cpro), the only proteolytic enzyme of the virus.

**Results:**

Individual expression of 3Cpro induced catalytic activity-dependent cell death, which was not abrogated by the pan-caspase inhibitor (z-VAD-fmk) and was not accompanied by phosphatidylserine externalization in contrast to other picornaviral 3C proteases. The cell survival was also not affected by the inhibitors of cysteine proteases (z-FA-fmk) and RIP1 kinase (necrostatin-1), critical enzymes involved in non-apoptotic cell death. A substantial fraction of dying cells demonstrated numerous non-acidic cytoplasmic vacuoles with not previously described features and originating from several types of endosomal/lysosomal organelles. The lysosomal protein Lamp1 and GTPases Rab5, Rab7, Rab9, and Rab11 were associated with the vacuolar membranes. The vacuolization was completely blocked by the vacuolar ATPase inhibitor (bafilomycin A1) and did not depend on the activity of the principal factors of endosomal transport, GTPases Rab5 and Rab7, as well as on autophagy and macropinocytosis.

**Conclusions:**

3Cpro, apart from other picornaviral 3C proteases, induces caspase-independent cell death, accompanying by cytoplasmic vacuolization. 3Cpro-induced vacuoles have unique properties and are formed from several organelle types of the endosomal/lysosomal compartment. The data obtained demonstrate previously undocumented morphological characters of the 3Cpro-induced cell death, which can reflect unknown aspects of the human hepatitis A virus-host cell interaction.

**Electronic supplementary material:**

The online version of this article (doi:10.1186/s12860-015-0050-z) contains supplementary material, which is available to authorized users.

## Background

3C proteases are the main proteolytic enzymes of picornaviruses. These enzymes catalyze the processing of polyproteins yielding intermediate and mature viral proteins (reviewed in [1]). In addition to this major function, 3C proteases can digest host cell proteins. The cleavage of transcription and translation factors [2-11], histones [12], cytoskeletal proteins [13,14], and cell antiviral immunity factors [15-17] by 3C proteases suppresses the host cell functions and induces its death. Individual expression of 3C proteases of enterovirus 71 [18], poliovirus [19], and Coxsackievirus [20] induces effects similar to those observed in viral infections. This advances these enzymes as critical cytotoxic factors of picornaviruses.

In all cases when the cytotoxic effect of 3C proteases was described, these enzymes induced cell death via the caspase-dependent apoptotic pathway [18-20]. No data on the cytotoxic effect of 3C protease of human hepatitis A virus (3Cpro), which is the only proteolytic enzyme on the virus, are currently available. At the same time, 3Cpro is known to digest host cell proteins of the same functional classes as other targets of 3C proteases. These proteins include poly(A)-binding protein (PABP) [21], poly(rC)-binding protein 2 (PCBP2) [22], and mitochondrial factors of cell innate immunity MAVS and TRIF [23,24]. Thus, the induction of apoptosis by 3Cpro similar to other 3C proteases could be expected.

However, this study presents the first demonstration that the 3C protease of human hepatitis A virus, unlike other picornaviral 3C proteases, induces caspase-independent cell death. The 3Cpro-induced cell death is accompanied by the accumulation of cytoplasmic vacuoles and depends on the enzyme catalytic activity. These vacuoles have unique properties and are formed from several organelle types of the endosomal/lysosomal compartment. The data obtained indicate that 3Cpro induces caspase-independent cell death with previously undocumented features.

## Results

### Expression of 3Cpro and its catalytically inactive variant

Monoclonal cell lines of human lung adenocarcinoma A549 Tet-Off Advanced and human lung carcinoma Calu-1 Tet-Off Advanced constitutively expressing transactivator protein tTA were established (referred to as A549 and Calu-1 below).

Plasmid pBI-EGFP was used as the expression vector. It drives the expression of target and reporter genes under the control of a tTA-responsive bidirectional promoter. Enhanced green fluorescent protein (eGFP) was used as a reporter to identify cells carrying the vector after transient transfection.

**Figure 1:**
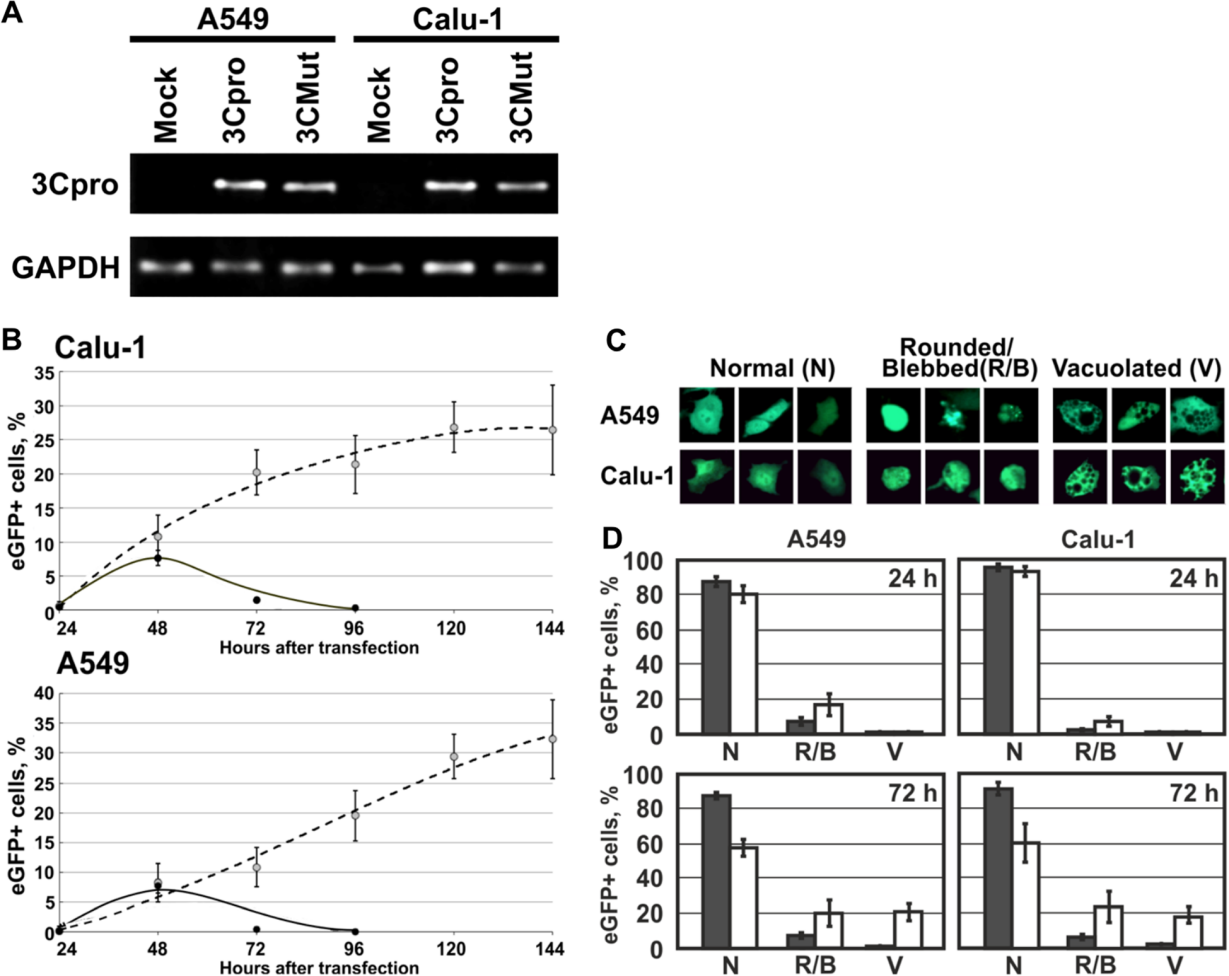
**Reverse PCR transcription analysis and dynamics of eGFP-positive cell accumulation in cultures. A**. Transcripts were detected 48 h after A549 and Calu-1 transfection with pBI-EGFP (Mock), pBI-EGFP/3C, encoding intact 3C protease (3Cpro), and pBI-EGFP/3CMut, encoding catalytically inactive 3C protease (3CMut). Glyceraldehyde 3-phosphate dehydrogenase (GAPDH) was used as a reference gene. **B**. The proportions of eGFP-positive cells were counted in A549 and Calu-1 cultures 144 h post-transfection (p.t.) with pBI-EGFP/3C (black points, solid line) and pBI-EGFP (light points, dotted line). Presented values were averaged for at least two independent experiments including four measurements each. The confidence interval was calculated at 95%. **C**. 3Cpro-expressing cells 48 h p.t. Normal (N), cells with normal morphology; Rounded/Blebbed (R/B), round and shrunk cells with smooth or blebbed plasma membrane; Vacuolated (V), vacuolated cells. **D**. Proportions of eGFP-positive cells of different morphology in control (gray bars) and experimental (empty bars) cultures. The values were averaged for at least two independent experiments. Two hundred cells were counted in three replicates for each time point. The confidence interval was calculated at 95%.

Protease 3C of human hepatitis A virus (3Cpro) was expressed in intact (pBI-EGFP/3C) and catalytically inactive (pBI-EGFP/3CMut) variants. The enzyme was inactivated by a Cys172 → Ala substitution, which was shown to suppress 3Cpro proteolytic activity [25]. The accumulation of 3Cpro transcripts was confirmed after the transfection of A549 and Calu-1 cell lines with pBI-EGFP/3C and pBI-EGFP/3CMut (Figure 1A).

### Individual expression of 3Cpro gene induces morphological changes and cell death

To investigate the effect of 3Cpro, A549 and Calu-1 cells were transiently transfected with pBI-EGFP/3C (A549/3Cpro and Calu-1/3Cpro) and pBI-EGFP lacking the 3Cpro gene (A549/Mock and Calu-1/Mock). Control cultures A549/Mock and Calu-1/Mock demonstrated gradual accumulation of eGFP-positive cells (Figure 1B). By the end of the observation period (144 h), their proportion amounted to about 30% of total culture cells. Most eGFP-positive cells retained the morphology typical for this cell line throughout the observation period (Figure 1C).

The proportion of eGFP-positive cells in experimental cultures A549/3Cpro and Calu-1/3Cpro reached the maximum 48 h p.t. (8-10%) and decreased to 1-2% 72 h post-transfection (p.t.). Only single eGFP-positive cells were observed 96 h p.t. (Figure 1B). A substantial fraction of eGFP-positive cells in experimental cultures demonstrated an altered morphology. There were cells that became round and shrunk, while their plasma membrane could remain smooth or became blebbed (Figure 1C). The morphology of these cells resembled that of apoptotic cells. In addition, there were cells that remained spread but contained a lot of cytoplasmic vacuoles. The proportion of different morphological types of eGFP-positive cells varied with time (Figure 1D). Twenty-four hours p.t., most cells retained normal morphology. Fourty-eight hours p.t., a significant fraction of cells (about 20%) became vacuolated. The proportion of vacuolated cells remained unaltered up to 72 h p.t. The majority of eGFP-positive cells had round shape 96 h p.t., while the cells with normal morphology could hardly be observed.

Time-lapse microscopy of the cell cultures at 5-min intervals for 96 h demonstrated that vacuolated cells remained spread even many hours after numerous vacuoles emerged. Shortly before detachment of vacuolated cells from the substrate, the vacuoles disappeared and cells became indistinguishable from the round and shrunk cells (see Additional file 1: Supplemental video).

The data obtained suggest that 3Cpro induces cell death. Death of a substantial fraction of cells was accompanied by the accumulation of cytoplasmic vacuoles. Finally, all 3Cpro-expressing cells before detachment acquired similar apoptotic-like morphology (Additional file 2: Figures S2 and S3).

### Catalytically inactive 3Cpro induces no cell vacuolization and death

Following transformation with pBI-EGFP/3CMut, the accumulation dynamics of eGFP-positive cells and their morphology were the same in experimental A549/3CMut and Calu-1/3CMut and control A549/Mock and Calu-1/Mock cultures (Additional file 2: Figure S3). Thus, cell vacuolization and death depend on the proteolytic activity of 3Cpro.

### 3Cpro-Induced cell death does not include caspase activation

The type of 3Cpro-induced cell death was evaluated by the state of the nuclei and chromatin, functional state of mitochondria, phosphatidylserine localization, and activation of caspases in 3Cpro-expressing cells.

**Figure 2:**
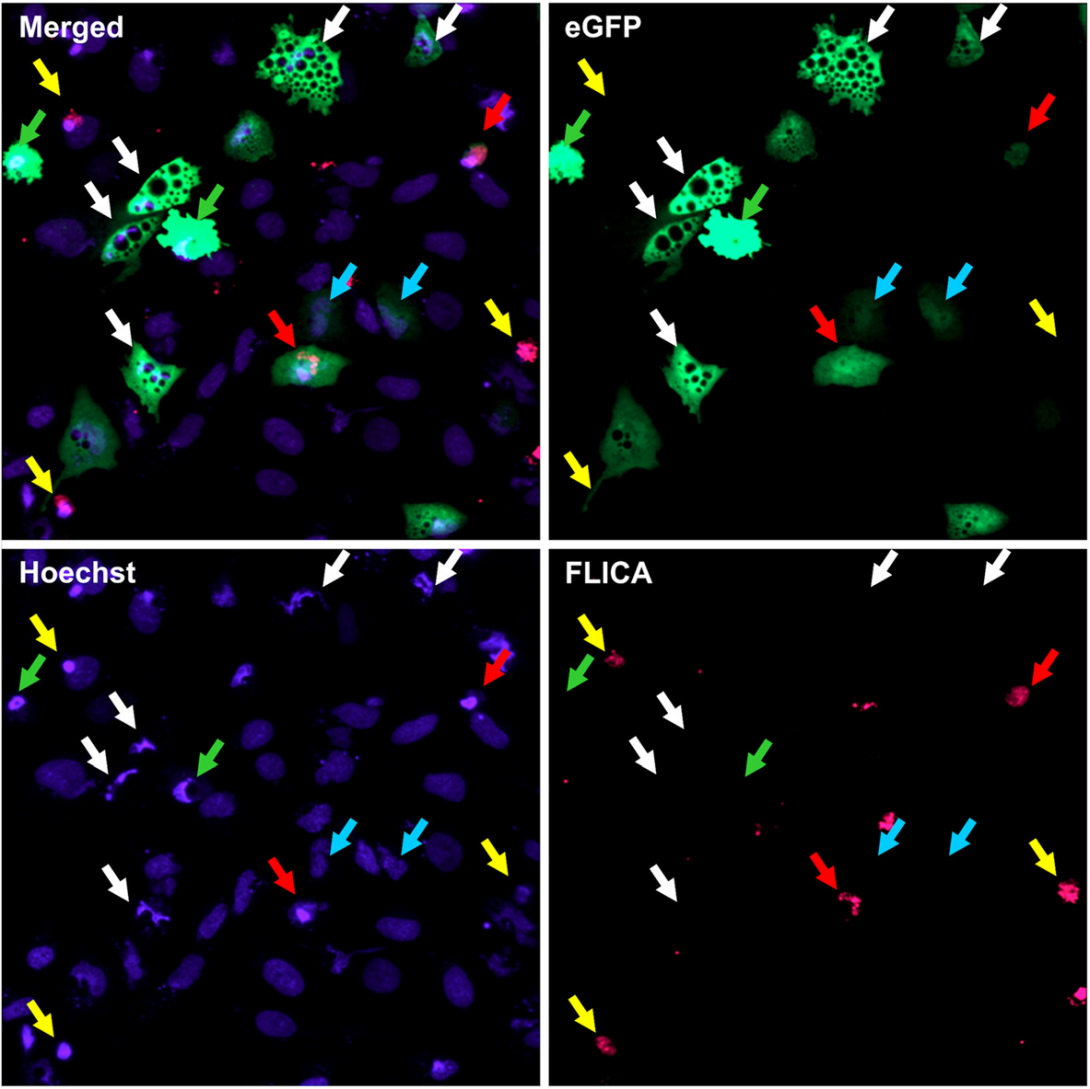
**Characteristics of 3Cpro-expressing cells.** Calu-1/3Cpro culture is shown 72 h p.t. Chromatin was visualized by DNA-intercalating dye Hoechst 33342 (**Hoechst** channel). Active caspases were detected by fluorescent agent FLICA (**FLICA** channel). eGFP-positive cells with normal morphology are indicated by cyan arrows. eGFP-positive vacuolated and round cells with condensed chromatin and inactive caspases are indicated by white and green arrows, respectively. eGFP-positive cells with active caspases are indicated by red arrows. Cells demonstrating chromatin condensation and karyorrhexis but not accumulating eGFP are indicated by yellow arrows. **eGFP** is the channel for GFP fluorescence; **Merged** channel shows overlapping signals.

Control A549/Mock and Calu-1/Mock cells had normal nuclear morphology and chromatin state throughout the observation period (72 h). Most round/shrunk A549/3Cpro and Calu-1/3Cpro cells demonstrated chromatin condensation and karyorrhexis 48 h p.t., while only some vacuolated cells showed nuclear deformation and partial chromatin condensation at the sites of contact with the vacuoles (data not shown). After 72 h, most vacuolated cells demonstrated pronounced chromatin condensation and their nuclei looked fragmented (Figure 2). At the same time, vacuolated cells demonstrated no plasma membrane blebbing and remained spread. Chromatin in vacuolated and round/shrunk cells was not stained by propidium iodide, which indicated their intact plasma membrane and non-necrotic death.

**Figure 3:**
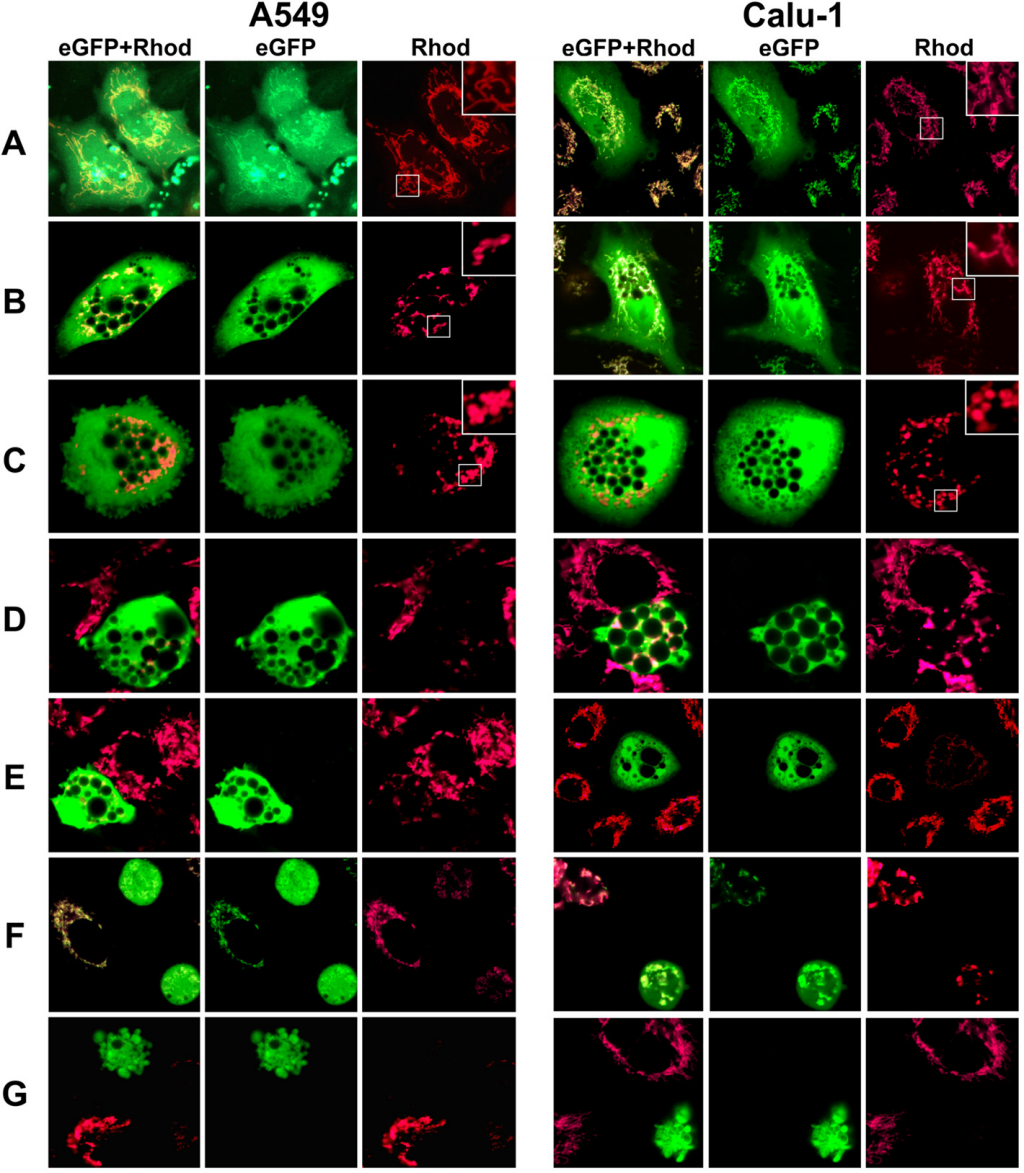
**Mitochondrial status in Calu-1 and A549 cells expressing 3Cpro.** Cells maintaining mitochondrial transmembrane potential were visualized using the potential-dependent fluorescent dye rhodamine 123. A549/Mock and Calu-1/Mock cells 48 h p.t. **(A)**. A549/3Cpro and Calu-1/3Cpro cells: vacuolated, 48 h p.t. **(B, C)** and 72 h p.t. **(D, E)**; round, 72 h p.t. **(F)**; with blebbed plasma membrane, 72 h p.t. **(G)**. EGFP and Rhod are channels for GFP and rhodamine 123 fluorescence, respectively; eGFP + Rhod channel shows overlapping signals.

Mitochondria were examined and their membrane potential (MMP) was evaluated using rhodamine 123 (Rh123), a potential-dependent fluorescent dye [26]. Throughout the observation period (72 h), mitochondria maintained their MMP and normal size and formed reticulum in control A549/Mock and Calu-1/Mock cells (Figure 3A). The majority of vacuolated cells in A549/3Cpro and Calu-1/3Cpro cultures demonstrated disrupted mitochondrial reticulum and swelling of MMP-maintaining mitochondria 48 h p.t. (Figure 3C). At the same time, certain vacuolated cells completely or partially retained their mitochondrial reticulum and normal mitochondrial size (Figure 3B). The presence of such cells suggests that initial vacuoles are formed before the changes in mitochondrial state become apparent. Vacuolated cells demonstrated a substantial decrease in the number of MMP-maintaining mitochondria 72 h p.t. At the same time, the intensity of mitochondrial staining by Rh123 in most vacuolated cells was lower than in untransformed cells (Figure 3D, E), which likely indicates decreased MMP. A similar pattern was observed for round cells with smooth plasma membrane (Figure 3F). No Rh123-positive mitochondria were observed in cells with blebbed plasma membrane (Figure 3G).

Chromatin condensation, karyorrhexis, decreased MMP, and reduced cell volume are markers of apoptosis, which is usually accompanied by phosphatidylserine exposure on the outer layer of the plasma membrane and induction of caspases [27]. However, phosphatidylserine was undetectable on the surface of A549/3Cpro and Calu-1/3Cpro cells (data not shown). The pan-caspase fluorescent reagent FLICA revealed only single cells with induced caspases 48 h and 72 h p.t. (Figure 2). Remarkably, all such cells were round, whereas no caspase activation was detected in vacuolated cells and cells with blebbing. Cell culturing with the caspase inhibitor z-VAD-fmk did not prevent vacuolization and had no notable effect on the survival of 3Cpro-expressing cells.

It should be noted that A549/3Cpro and Calu-1/3Cpro as well as A549/Mock and Calu-1/Mock cultures included a minor fraction of eGFP-negative round cells demonstrating chromatin condensation, karyorrhexis, and caspase activation, which indicate their death through caspase-dependent apoptosis (Figure 2). The capacity of both cell lines to follow the caspase-dependent pathway has been additionally demonstrated using the standard apoptosis-inducing drug doxorubicin (data not shown).

The data obtained demonstrate that 3Cpro induces caspase-independent cell death in both studied lines despite their susceptibility to caspase-dependent apoptosis.

### Inhibitors of intracellular cysteine proteases and RIP1 kinase do not suppress 3Cpro-induced cell death

Cytoplasmic vacuolization accompanies alternative non-apoptotic cell death pathways. Some of them are driven by cysteine proteases cathepsins and calpains [28-31], which can substitute caspases in their absence [32-37]. However, the incubation of A549/3Cpro and Calu-1/3Cpro cells with the inhibitor of lysosomal cysteine proteases Z-FA-fmk neither prevented vacuolization nor had a notable effect on the survival of transfected cells (Additional file 2: Figures S2 and S3). A similar pattern was observed for the caspase inhibitor Z-VAD-fmk, high concentrations of which (over 10 μM) block cysteine cathepsins and calpains apart from caspases [38,39]. Apparently, cathepsins and calpains as well as caspases are not solely responsible for 3Cpro-induced cell death.

Necroptosis, the best studied subtype of programmed necrosis, is predominantly mediated by RIP1 [40]. However, the specific RIP1 inhibitor necrostatin-1 [30,41] had no effect on the vacuolization and survival of A549/3C and Calu-1/3C cells either (Additional file 2: Figures S2 and S3).

### 3Cpro-induced vacuoles are bounded by a single bilayer and contain multimembrane structures

**Figure 4:**
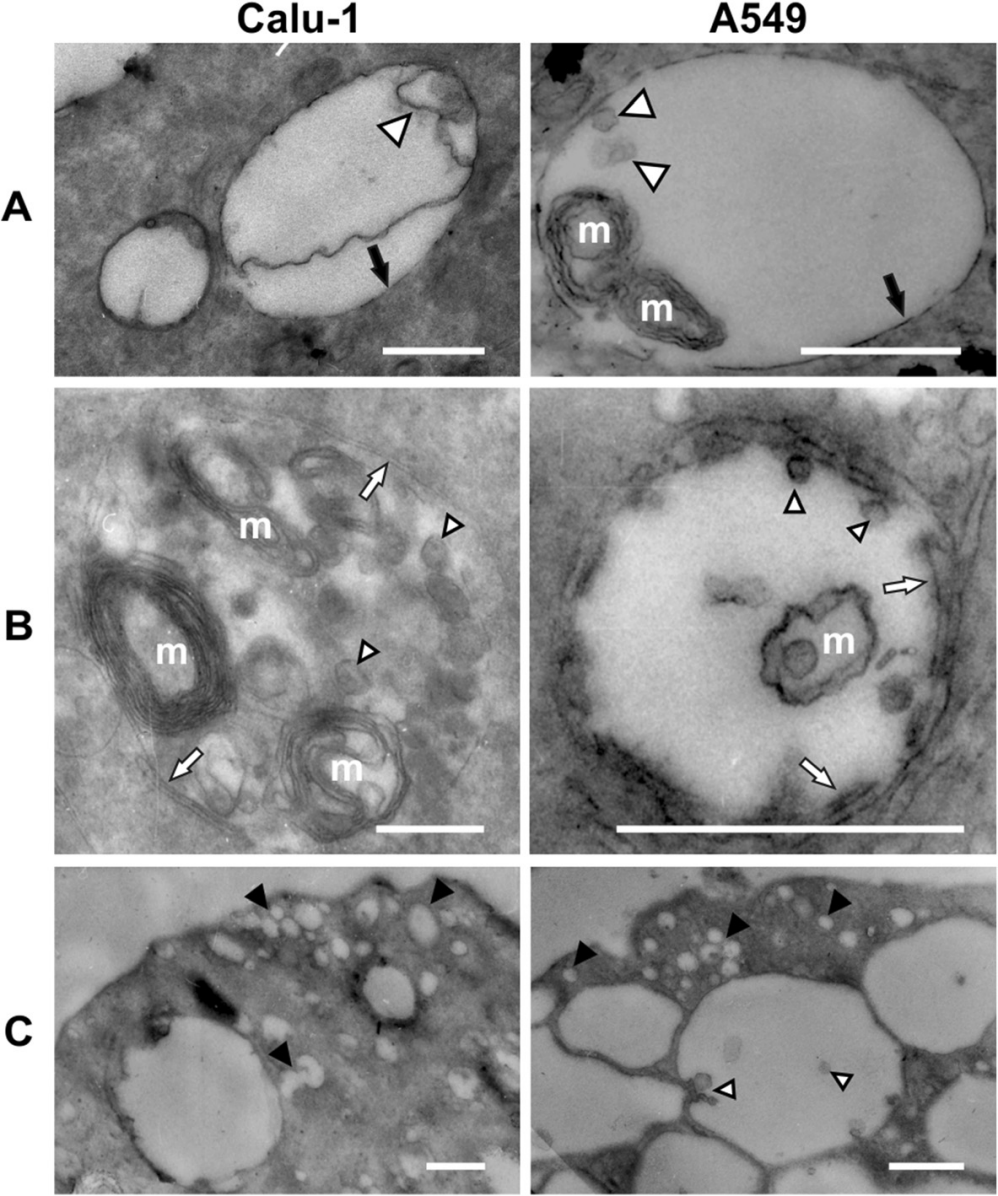
**Ultrastructural analysis of 3Cpro-induced vacuoles.** Cytoplasmic vacuoles in A549/3Cpro and Calu-1/3Cpro cells were analyzed by electron microscopy. **A**. Vacuoles bounded by a single membrane layer. **B**. Vacuoles with blurred boundaries. **C**. Vesicles neighboring vacuoles. Vesicles inside vacuoles are indicated by triangles; multimembrane formations, m; small vesicles neighboring vacuoles, *. Scale, 0.5 μm.

Ultrastructural analysis using electron microscopy demonstrated that cytoplasmic vacuoles are bounded by a single bilayer membrane in A549/3Cpro and Calu-1/3Cpro cells (Figure 4A). A portion of vacuoles had no clear boundary and were surrounded by structures resembling endoplasmic reticulum (ER) or Golgi cisternae (Figure 4B). Certain vacuoles contained multimembrane structures and small vesicles; however, the majority of vacuoles contained no significant quantities of membranous material (Figure 4A, B). The vacuoles often neighbored smaller vesicles also bounded by a single membrane layer and largely containing no membranous inclusions (Figure 4C). Such vesicles were also numerous in the vicinity of the plasma membrane, which points to endocytosis as the mechanism of their formation. Morphological analysis suggests that the vacuoles might be formed from components of the endosomal/lysosomal, ER, or Golgi compartments.

### Mitochondria, endoplasmic reticulum, and Golgi are not involved in vacuole formation

**Figure 5:**
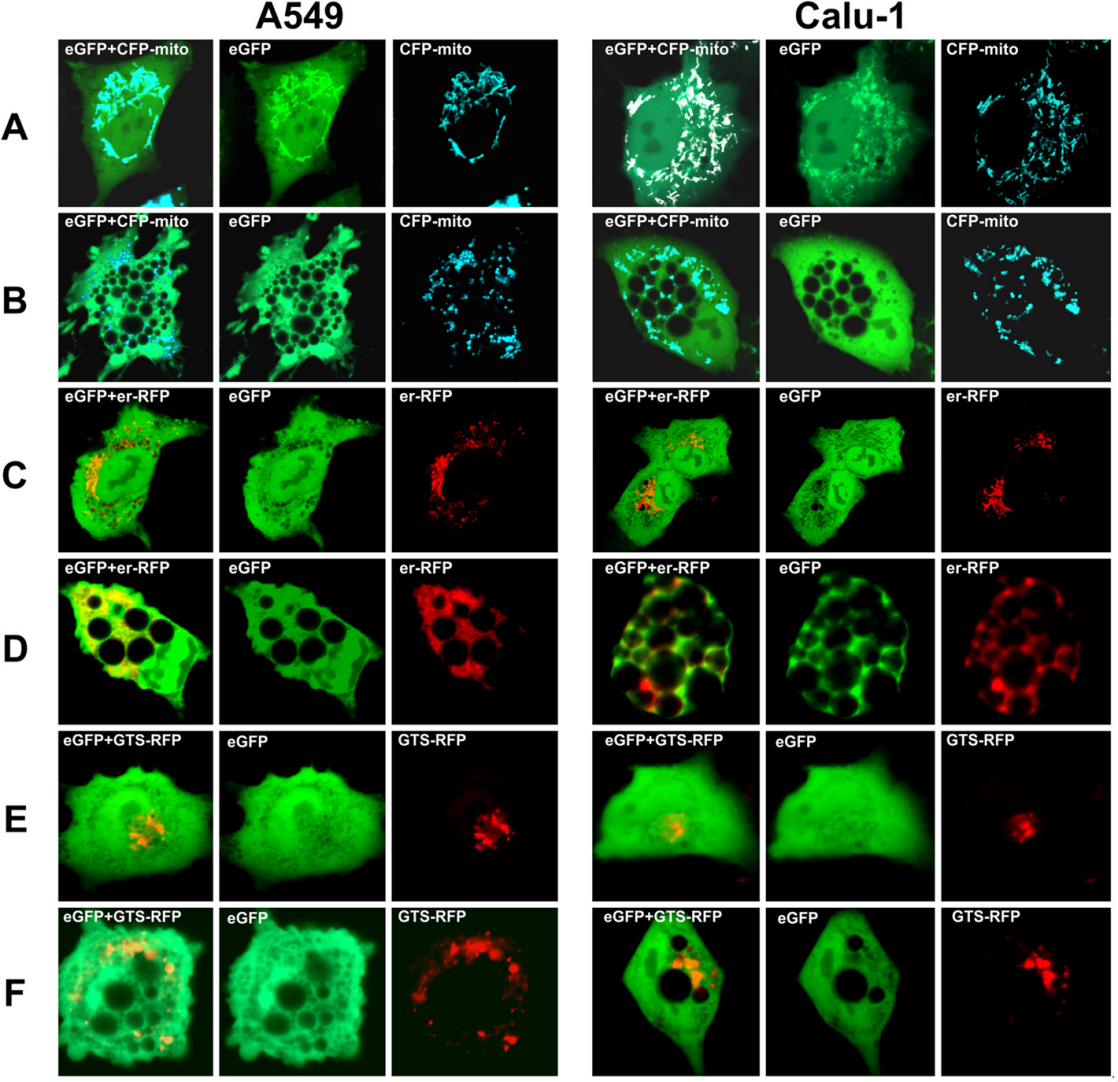
**Statuses of mitochondria, endoplasmic reticulum, and Golgi in vacuolated cells.** Localization of fluorescent proteins targeted to mitochondria (CFP-mito), endoplasmic reticulum (er-RFP), and trans-Golgi network (GTS-RFP) in control A549/Mock, Calu-1/Mock **(A,** **C,** **E)** and vacuolated A549/3Cpro, Calu-1/3Cpro **(B,** **D,** **F)** cells, respectively.

The origin and properties of cytoplasmic vacuoles were further studied using a set of vectors encoding fluorescent proteins targeted to different cellular compartments. Mitochondria were studied using a cyan fluorescent protein fused to the mitochondrial targeting signal from human cytochrome c oxidase subunit VIII (CFP-mito), which visualizes mitochondrial membrane irrespective of the functional state of these organelles [42]. Mito-CFP accumulated in mitochondria of 3Cpro-expressing cells, but was not observed in vacuolar membranes or lumen (Figure 5A, B).

ER was visualized using a red fluorescent protein fused to the ER retention signal SEKDEL (er-RFP) [43]. In control A549/Mock and Calu-1/Mock cells, er-RFP was found in the granules characteristic for ER (Figure 5C). In vacuolated A549/3Cpro and Calu-1/3Cpro cells er-RFP demonstrated homogeneous cytoplasmic localization but was not found in vacuolar lumen and membranes (Figure 5D). A similar pattern was observed for staining by the low-molecular-weight ER-Tracker Blue-White DPX dye specific for ER membranes (data not shown). The data obtained indicate abnormal ER function and its possible degradation.

Components of the trans-Golgi network were visualized using RFP with the Golgi retention signal of human β-1,4-galactosyltransferase. In both control culture cells and vacuolated ones, this fluorescent protein accumulated in distinct organelles but not in the vacuolar lumen and membranes (Figure 5E, F).

Thus, the data obtained suggest that the vacuoles originate from organelles other than mitochondria, ER, and trans-Golgi network.

### Vacuoles have endosomal/lysosomal origin

**Figure 6:**
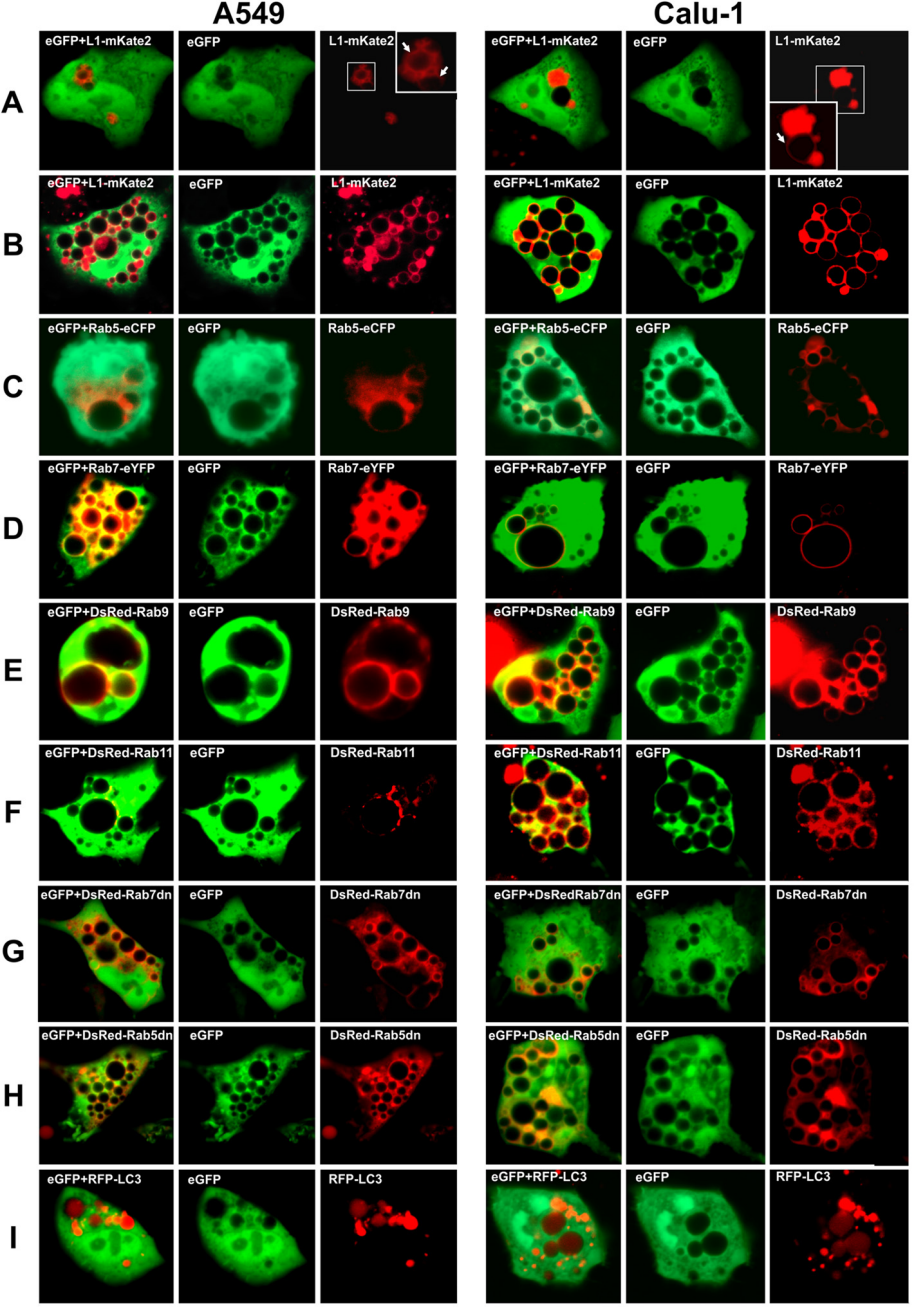
**Origin of 3Cpro-induced vacuoles.** Vacuolated A549/3Cpro and Calu-1/3Cpro cells expressing fusion proteins L1-mKate2 48 h p.t. **(A)** and 72 h p.t. **(B)**, Rab5-eCFP **(C)**, Rab7-eYFP **(D)**, DsRed-Rab9 **(E)**, DsRed-Rab11 **(F)**, DsRed-Rab7dn **(G)**, DsRed-Rab5dn **(H)**, and LC3-RFP **(I)** 72 h p.t. Dominant-negative mutants of GTPases are indicated by “dn”.

Lysosomes and late endosomes were visualized using the lysosomal-associated membrane protein 1 (Lamp1) fused with the fluorescent protein mKate2 (L1-mKate2). L1-mKate2 localized in vesicles dispersed or clustered in the cytoplasm of control A549/Mock and Calu-1/Mock cells 48 h p.t. Three cell types could be recognized in A549/3Cpro and Calu-1/3Cpro cells. Type I cells had no vacuoles and the localization of L1-mKate2 was similar to that in control cultures. Type II cells had single small L1-mKate2-positive vacuoles associated with clusters of L1-mKate2-positive vesicles (Figure 6A). Type III cells contained numerous vacuoles and L1-mKate2 localized to the membranes of all vacuoles (Figure 6B). Notice that the cytoplasm of vacuolated cells contained far less L1-mKate2-positive vesicles compared to vacuole-free cells. Overall, the data obtained indicate that the vacuoles originate from organelles of the endosomal/lysosomal compartment and that clusters of these organelles can be the sites of vacuole formation.

Normally Lamp1 goes through early endosomes on the way to late endosomes and lysosomes [44]. Since 3Cpro induces abnormal function of the endosomal/lysosomal compartment, mislocalization of L1-mKate2 cannot be excluded. Hence, the observed L1-mKate2 localization cannot unambiguously point to the organelle type of the endosomal/lysosomal compartment that gave rise to the vacuoles. In this context, cytoplasmic GTPases of the Rab family specifically associated with membranes of different endosome types [45] were used to visualize individual organelle populations. Fusion proteins Rab5-eYFP, Rab7-eCFP, DsRed-Rab9, and DsRed-Rab11 served as markers of early endosomes, late endosomes/lysosomes, and endosomes recycling to the trans-Golgi network and plasma membrane, respectively [46,47]. In vacuolated A549/3Cpro and Calu-1/3Cpro cells, all these markers accumulated in the vacuolar membranes. In addition, the vacuolar membrane-associated vesicles with the fusion proteins have been revealed (Figure 6C-F). At the same time, the overexpression of these proteins *per se* in control A549/Mock and Calu-1/Mock cells induced no vacuole formation or other morphology alterations (data not shown).

It should be noted that the incubation of A549/3Cpro and Calu-1/3Cpro cells with colchicine, an inhibitor of polymerization of microtubules that mediate the transport of organelles of the endosomal compartment, did not suppress vacuole formation (data not shown). Thus, 3Cpro-induced vacuole formation does not depend on the microtubular activity.

The data obtained indicate that several organelle types of the endosomal/lysosomal compartment are involved in the vacuole formation.

### Overexpression of dominant-negative Rab5 and Rab7 does not suppress vacuole formation

The relationship between 3Cpro-induced vacuolization and Rab5 and Rab7 functions was evaluated using their dominant-negative mutants Rab5/N133I (unable to bind GTP [48]) and Rab7/T22N (constitutively GDP-bound [49,50]) fused with the fluorescent protein DsRed. The expression level of these GTPases evaluated from DsRed fluorescence intensity varied significantly from cell to cell. Accordingly, the cells demonstrating top fluorescence levels were selected for analysis.

A549/3Cpro and Calu-1/3Cpro cells with high levels of Rab5/N133I and Rab7/T22N proved to contain the vacuoles, and both GTPases were associated with the vacuolar membranes (Figure 6G, H). The size and morphology of these vacuoles was indistinguishable from those in cells expressing 3Cpro alone.

### Autophagy is not essential for 3Cpro-induced vacuolization and cell death

The role of autophagosomes in the 3Cpro-induced vacuolization was evaluated using the LC3 protein (specific for these organelles) fused to fluorescent protein mRFP. The fusion protein was not accumulated in the membranes but localized diffusely in the vacuolar lumen (Figure 6I). This indicates the involvement of autophagosomes in vacuole formation. Autophagosome-mediated formation of vacuoles is observed after using some agents that impair autophagy. In some cases, such impairments proved to result from the constitutive activation of the ERK1/2 signaling pathway [51,52]. However, the incubation of 3Cpro-expressing cells with the inhibitors of this pathway (PD98059 and Sc-353669) did not suppress the vacuolization and had no noticeable effect on cell survival. Likewise, no noticeable effect was observed after cell exposure to 3-methyladenine, an inhibitor of class 3 phosphatidylinositol 3-kinase and autophagosome formation (Additional file 2: Figures S2 and S3). Thus, the data obtained indicate that the 3Cpro-induced vacuolization and cell death do not depend on autophagy.

### Vacuolization is not essential for 3Cpro-induced cell death

**Figure 7:**
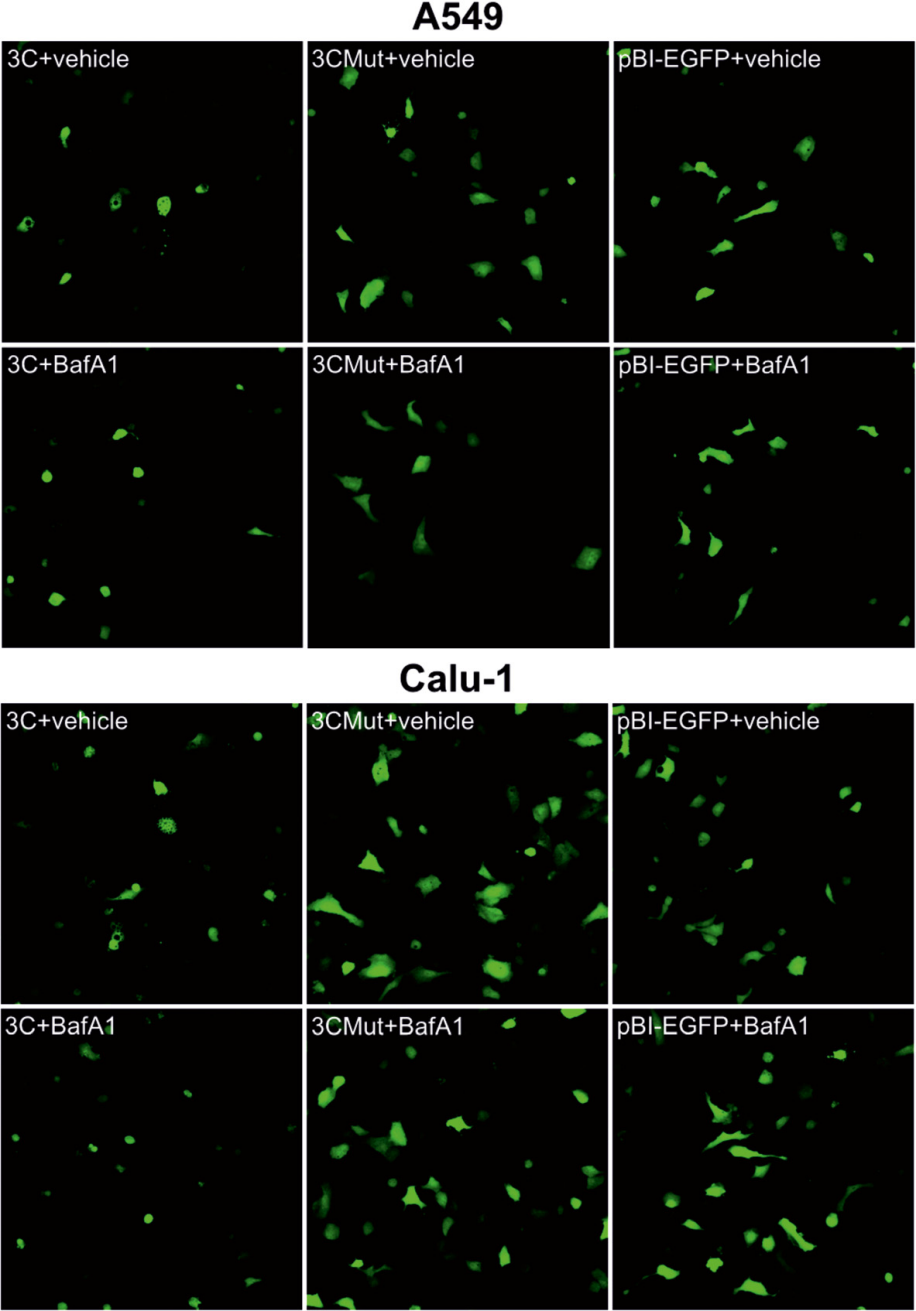
**Effect of Bafilomycin A1 on vacuolization.** A549 and Calu-1 cells transfected with pBI-EGFP (Mock), pBI-EGFP/3C (3C) or pBI-EGFP/3Cmut (3Cmut) and treated by Bafilomycin A1 (BafA1) or equal quantity of vehicle (DMSO) 48 h p.t.

Cell incubation with the inhibitor of vacuolar ATPase bafilomycin A1 (BafA1), which is often used to suppress autophagy [53-55], completely blocked the vacuolization but had no effect on cell death (Figures 7, Additional file 2: Figure S3). Since BafA1 blocks not only autolysosome formation but also endosome fusion [56,57], this finding in the context of no effect of 3-methyladenine indicates again that the vacuolization results from the fusion of organelles of the endosomal/lysosomal compartment. The effect of BafA1 suggests another important conclusion: the vacuolization event is not essential for 3Cpro-induced cell death.

### 3Cpro-induced vacuoles do not have properties of degradative organelles

The 3Cpro-induced vacuoles carry markers of degradative organells that normally have acidic content and contain active hydrolases [58]. We tested if the vacuoles have the properties of degradative organelles using fluorescent substrate of cathepsin B (Magic Red) and pH-dependent dye (Neutral Red).

**Figure 8:**
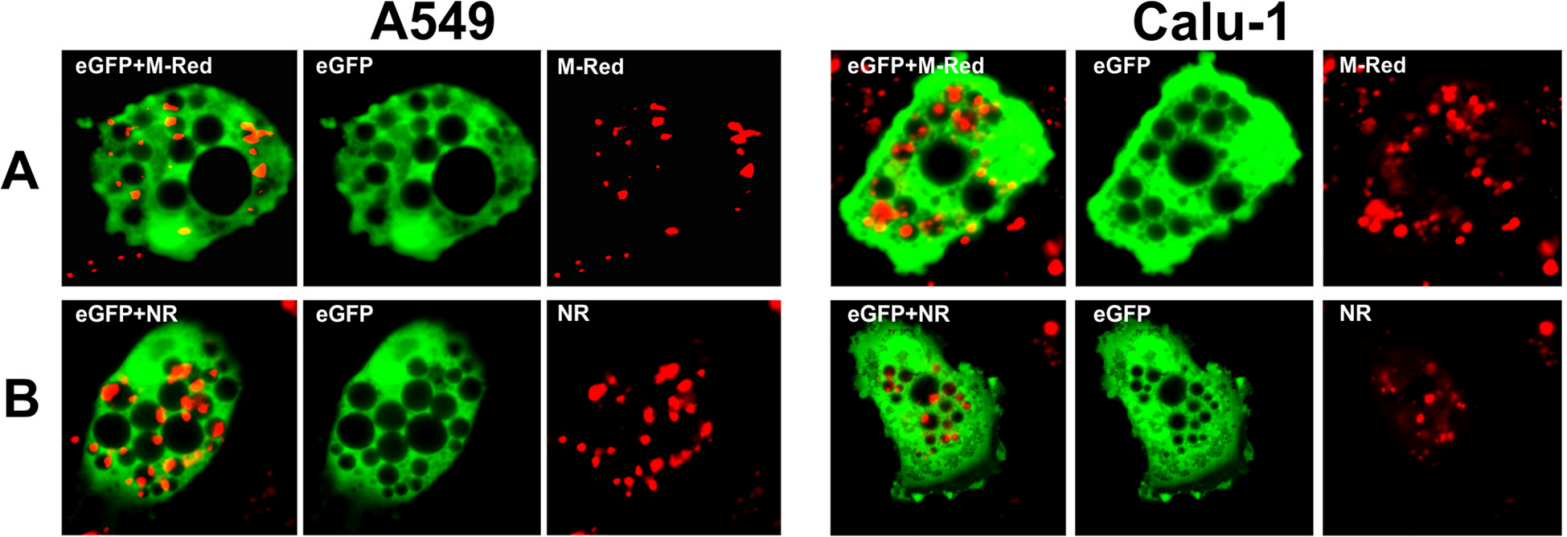
**Cathepsin B activity and localization of acidic organelles in vacuolated cells.** A549/3Cpro and Calu-1/3Cpro cells 72 h p.t. after incubation with the fluorescent substrate of cathepsin B Magic Red (M-Red) **(A)** and pH-dependent fluorescent dye Neutral Red (NR) **(B)**.

In all vacuolated cells, the fluorescent product of Magic Red hydrolysis was detected in individual vesicles, most of which are localized within the vacuoles (Figure 8A). It was not detected in the vacuolar lumen and cytoplasm of vacuolated cells. This suggests that active lysosomal proteases are not released to the cytoplasm as observed in certain types of caspase-independent cell death. Cell staining with Neutral Red demonstrated that the vacuolar lumen is not acidic. At the same time, acidic vesicles were observed within the vacuoles (Figure 8B). Most likely, these vesicles contained active cathepsin B. It is of interest that the vesicles with the fluorescent product of Magic Red hydrolysis and acidic content were largely observed in smaller vacuoles and were nearly always absent in larger ones. At the same time, such vesicles were missing or sporadic in cells with numerous large vacuoles. Assuming that larger vacuoles have longer lifetime one can propose that the intravacuolar vesicles gradually lose their degradative properties.

### Vacuolization is not a consequence of macropinocytosis hyperstimulation

Vacuoles lacking the properties of degradative organelles and accumulating Lamp1 and Rab7 in their membranes are observed during cell death resulting from hyperstimulation of macropinocytosis. This cell death pathway was called methuosis [59].

**Figure 9:**
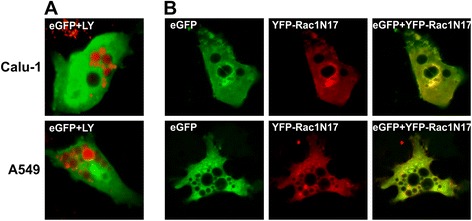
**Accumulation of Lucifer Yellow and Rac1(N17) in vacuolated cells.** A549/3Cpro and Calu-1/3Cpro cells 72 h p.t. after incubation with fluorescent dye Lucifer Yellow (LY) **(А)** or co-expressing dominant-negative mutant of GTPase Rac1(N17) **(B)**.

Macropinocytic activity was evaluated in A549/3Cpro and Calu-1/3Cpro cells using fluorescent dye Lucifer Yellow (LY), which cannot penetrate the cell membrane. LY could be detected in individual vacuoles of single vacuolated cells incubated with the dye for one or several hours 48 or 72 h p.t. Cell incubation for 12 h led to LY accumulation in vacuoles of all vacuolated cells (Figure 9A). These data indicate that the vacuoles can accumulate extracellular fluid. At the same time, the low rate of extracellular fluid accumulation contradicts possible hyperstimulation of macropinocytosis when detectable LY quantities are accumulated in vacuoles in 10–15 minutes [59].

The constitutive induction of GTPase Rac1 is a prerequisite for the hyperstimulation of macropinocytosis in methuosis [60]. Rac1 inhibition or overexpression of dominant-negative Rac1(N17) prevents macropinocytosis hyperstimulation and methuosis [60]. In our case, the expression of the fusion protein YFP-Rac1(N17) did not prevent the vacuolization even in cells with high YFP-Rac1(N17) levels (Figure 9B). Similarly, incubation of A549/3Cpro and Calu-1/3Cpro cells with filipin, which prevents the formation of macropinosomes [61], had no effect on the size and number of vacuoles, as well as on cell viability (Additional file 2: Figures S2 and S3).

Overall, the data obtained suggest that 3Cpro-induced vacuolization is not a consequence of macropinocytosis hyperstimulation and that the cell death does not follow the methuosis pathway.

## Discussion

This study presents the first demonstration that 3C protease of human hepatitis A virus can induce cell death dependent on the enzyme proteolytic activity and accompanied by the formation of cytoplasmic vacuoles through the fusion of organelles of the endosomal/lysosomal compartment.

Individual expression of many picornaviral proteins [62] including 3C proteases of enterovirus 71 [18], poliovirus [19], and Coxsackievirus [20] was shown to induce cell death. In all described cases, 3C protease-induced cell death depended on the enzyme activity and was recognized as caspase-dependent apoptosis according to both morphological and biochemical indications.

Cell death induced by 3C protease of human hepatitis A virus (3Cpro) also showed some properties typical for apoptosis: disruption of the mitochondrial reticulum, swelling of mitochondria, loss of mitochondrial membrane potential, chromatin condensation, and karyorrhexis at the background of plasma membrane integrity. (It is of note that the dying cells with vacuoles demonstrated chromatin condensation and karyorrhexis long before lost of spreading, which is not typical for apoptosis when the changes in nuclear morphology are usually observed after lost of spreading and shrinking [63,64]). Considering that the nuclear fragments were sandwiched between the vacuoles, one can propose that the mechanical impact of vacuoles could induce the changes in nuclear morphology. 3Cpro-induced cell death was not accompanied by phosphatidylserine externalization and caspase activation; likewise, it was not blocked by the pan-caspase inhibitor z-VAD-fmk. A similar set of characters has been previously reported for alternative non-apoptotic cell death types [65-68]. Thus, 3Cpro induces caspase-independent cell death unlike other picornaviral 3C proteases.

A significant fraction of cells undergoing 3Cpro-induced death demonstrated cytoplasmic vacuolization before mitochondrial depolarization and other cell death signs. Vacuolization of different intracellular compartments is a marker of certain pathological states [69-75] and accompanies caspase-independent cell death [30,27,52,76-78]. Vacuoles are formed from different organelles in different types of caspase-independent cell death, which allows us to use the properties and origin of vacuoles to identify cell death type.

We have shown that the 3Cpro-induced vacuolization did not affect endoplasmic reticulum, mitochondria, and Golgi and was not blocked by necrostatin-1, an inhibitor of RIP1 kinase. This allowed us to exclude the paraptotic and necroptotic pathways [30,79,80].

The factors of cell death and cytoplasmic vacuole formation include autophagy abnormalities [81-84]. The lumen of 3Cpro-induced vacuoles proved to contain LC3 protein, which testifies to the involvement of autophagosomes in the formation of vacuoles. Cell death and vacuolization associated with abnormal autophagy can result from the constitutive activation of the Raf-MEK-ERK1/2 cascade [51,52]. Nevertheless, the used inhibitors of MEK kinases had no effect on cell vacuolization and death. The suppression of autophagy by 3-methyladenine (which blocks autophagosome formation) also had no effect on the action of 3Cpro. At the same time, the inhibitor of vacuolar ATPase bafilomycin A1 (BafA1) completely blocked the vacuolization but not the cell death. Although BafA1 is commonly used autophagy suppressor, it also inhibits fusion of endocytic organelles [55,85]. In the context of the 3-methyladenine effect, the action of BafA1 is likely mediated by the inhibition of endocytosis pathway rather than autophagy suppression. Thus, the data obtained demonstrate that autophagy is not essential for 3Cpro-induced cell vacuolization and death, and vacuolization is a morphological indication but not the cause of 3Cpro-induced cell death.

The vacuolar membranes simultaneously accumulated markers of different types of endocytic organelles, fluorescent proteins fused with the Lamp1 sorting signal or with GTPases Rab5, Rab7, Rab9, and Rab11. Under normal homeostatic conditions, the cells have organelles simultaneously containing Rab7 and Rab9 (late endosomes), Rab7 and Lamp1 (late endosomes and lysosomes), as well as Rab5 and Rab7 (early endosomes); Rab11-containing endosomes usually represent a separate population [86-91]. Thus, 3Cpro-induced vacuoles are formed from several organelle types of the endosomal/lysosomal compartment.

The vacuolization of endocytic organelles has been shown previously in the following cases. The inhibition of kinases hVPS34 and PIKfyve, which regulate vesicular transport and sorting, leads to the vacuolization of late endosomes but does not involve other organelles [92-94]. The vacuoles induced by certain bacterial toxins, e.g., VacA from *Helicobacter pylori,* epsilon toxin from *Clostridium perfringens*, and CARDS toxin from *Mycoplasma pneumoniae*, also have an endosomal/lysosomal origin [50,95,96]. However, the vacuolization involves a single (at most two) endosomal type in all known cases. Thus, 3Cpro induces the formation of vacuoles with not previously described properties, and all major types of endocytic organelles are involved.

GTPases of the Rab family revealed in the vacuolar membranes have many functions in the homeostasis regulation in the endosomal/lysosomal compartment including hetero- and homotypic fusion of endosomes [46,47]. High levels of constitutively active forms of Rab5 and Rab7 are known to induce fusion and vacuolization of early and late endosomes, while the prevalence of their nonfunctional forms conversely blocks endosomal fusion [97,98]. Activity of Rab GTPases is required for the vacuolization process of endosomal/lysosomal organelles induced by certain bacterial toxins [50,95,96]. At the same time, 3Cpro-induced vacuolization is not suppressed by the overexpression of nonfunctional Rab5 or Rab7 variants, and thus does not depend on the function of these GTPases.

Some properties of 3Cpro-induced vacuoles (accumulation of extracellular fluid, non-acidic content, and markers of late endosomes and lysosomes in their membrane) draw them together with the vacuoles resulting from hyperstimulation of macropinocytosis in methuosis [59]. The formation of giant macropinosomes in methuosis requires the constitutive activation of GTPase Rac1 and is prevented by the overexpression of its nonfunctional variant or in the presence of filipin, an inhibitor of clathrin-independent endocytosis [99]. However, the overexpression of nonfunctional Rac1 or cell incubation with filipin did not prevent 3Cpro-induced cell vacuolization and death. Hence, 3Cpro-induced vacuolization does not depend on macropinocytic activity and cell death does not follow the methuosis pathway.

Overall, 3Cpro-induced vacuoles have previously undescribed features, and thus 3Cpro-induced cell death cannot be assigned to any currently known type of caspase-independent cell death accompanied by vacuolization.

In our opinion, it is of primary interest if the effects of 3Cpro described in this work are observed in cells infected by human hepatitis A virus. Apoptotic cell death was observed in studied cases of infection by cytopathogenic forms of the virus [100-102]. Apparently, 3Cpro is not the main cytotoxic factors in these cases. At the same time, a number of viruses can induce either apoptotic or caspase-independent cell death depending on infection conditions. For instance, caspase-independent cell death is triggered in abortive poliovirus infection [103,104] or at high infectious dose of West Nile virus [105]. Accordingly, 3Cpro can become the main cytotoxic factor under certain infection conditions.

The cytopathic effect of many viruses is manifested as specific changes in cellular compartments preceding cell death [106,107]. Morphological changes of cells infected with cytopathogenic forms of hepatitis A virus and other picornaviruses include swelling of ER cisternae and formation of multilayer membrane structures, vesicular structures, and cytoplasmic vacuoles [108-112]. Certain cytopathic effects of hepatitis A virus are due to viral proteins 2B, 2C, and 2BC [62,111,113]. At the same time, no data on the factors underlying the emergence of cytoplasmic vacuoles are currently available. The results obtained in this work allow us to propose that 3Cpro mediates the development of cytopathic cell morphology and the formation of vacuoles in hepatitis A virus infection. The involvement of picornaviral 3C proteases in cytopathic vacuolization has not been reported previously.

At the same time, a variety of proteins of other viruses can induce vacuolization. Most of them have no enzyme activity, e.g., large surface protein of hepatitis B virus [114,115], Env protein of murine leukemia virus [116-118], capsid protein VP1 of Simian vacuolating virus 40 [119], oncoproteins E5 and E6 of human papillomavirus [120-122], and A38L protein of vaccinia virus [123]. On the other hand, the vacuolization effect was described for NS3 proteases of certain flaviviruses. (Flaviviruses and picornaviruses belong to single-stranded positive-sense RNA viruses, and NS3 and 3Cpro proteases are assigned to the chymotrypsin structural family [124,125]). Cytoplasmic vacuolization is typical for flaviviral infections by hepatitis C virus [126], West Nile virus [105], and Dengue virus [127] and is also observed after individual expression of their NS3 proteases [128-130]. The case of bovine viral diarrhea virus (BVDV) is of particular interest since the infection-induced vacuoles have been characterized. The properties of vacuoles induced by BVDV and 3Cpro are similar: both originate from endosomal/liposomal organelles, have nonacidic content, and their formation is autophagy-independent [131]. Note that cytoplasmic vacuolization was observed only after infection with a cytopathogenic biotype, which differs from non-cytopathogenic one by elevated expression of protease NS3 [132,133].

Thus, analysis of published and obtained data indicates that the observed effect of 3Cpro can reflect the participation of this enzyme in the development of cytopathic morphology of infected cells. In this context, the mechanism of 3Cpro impact on the endosomal/lysosomal compartment and the role of this protease in the cytopathic effect of the human hepatitis A virus require further investigation.

## Conclusions

Analysis of the cytotoxic effect of 3C protease of human hepatitis A virus allowed us to demonstrate 3Cpro-induced cell death independent of caspases and accompanied by accumulation of cytoplasmic vacuoles. The cytotoxic and vacuolization effects of 3Cpro depend on its catalytic activity. 3Cpro-induced vacuoles have unique properties and originate from several organelle types of the endosomal/lysosomal compartment. The data obtained indicate that 3Cpro induces caspase-independent cell death with previously unreported morphological characters.

## Methods

### Materials

DMEM/F-12 and OptiMEM media, fetal bovine serum (FBS), phosphate-buffered saline (PBS), Lipofectamine 2000, ER-Tracker Blue-White DPX, and Image-iT LIVE Red Poly Caspases Detection Kit were purchased from Invitrogen (USA). Annexin V-Cy3 Apoptosis Detection Kit, G418 (Geneticin), bafilomycin A1, 3-methyladenine, filipin, PD98059, z-VAD-fmk, z-FA-fmk, necrostatin-1, propidium iodide (PI), rhodamin 123 (Rh123), Hoechst 33258, Lucifer Yellow, colchicine, and glutamine were bought from Sigma (Germany). Sc-353669 was from Santa Cruz Biotechnology (USA). Magic Red Cathepsin B Assay Kit was from Immunochemistry Technologies (USA). High Capacity cDNA Reverse Transcription Kit was purchased from Applied Biosystems (USA). RNAqueous Kit was from Ambion. RQ1 DNAse was purchased from Promega (USA). Plasmids and primers used in this study are described in Tables 1 and 2.

**Table 1:** **Plasmids used**

**Plasmid**	**Description**	**Reference/Source**
**Vectors encoding organelle-targeted fluorescent proteins**		
pmKate2-lyso	encodes mKate2 N-terminally fused to rat Lamp-1	Evrogen (Russia)
pTagCFP-mito	encodes TagCFP N-terminally fused to mitochondrial targeting sequence derived from the subunit VIII of human cytochrome C oxidase	Evrogen (Russia)
pTagRFP-Golgi	encodes TagRFP N-terminally fused to Golgi targeting sequence of human β-1,4-galactosyltransferase	Evrogen (Russia)
pRab7-EYFP^a^	encodes Rab7 C-terminally fused to eYFP	[88]
pRab5-ECFP^a^	encodes Rab5 C-terminally fused to eCFP	[88]
pDsRed-Rab5/DN^b^	encodes dominant-negative Rab5/N133I N-terminally fused to DsRed	[134,135]/Add #13051^g^
pDsRed-Rab7/DN^b^	encodes Rab7/T22N N-terminally fused to DsRed	[134,135]/Add #12662^g^
pDsRed-Rab9^b^	encodes Rab9 N-terminally fused to DsRed	[134,135]/Add #12677^g^
pDsRed-Rab11^b^	encodes Rab11 N-terminally fused to DsRed	[134,135]/Add #12679^g^
pmRFP-LC3^c^	encodes autophagosome-specific LC3 protein N-terminally fused to mRFP	[136]/Add #21075^g^
pYFP-Rac1(N17)^d^	encodes Rac1/T17N N-terminally fused to fluorescent protein YFP	[137]/Add #11395^g^
pER-RFP^e^	encodes RFP N-terminally fused to CD5 leader sequence and C-terminally fused to SEKDEL amino acid sequence	[43]
**Vectors for tet-off advanced expression system**		
pTet-Off Advanced	encodes the tetracycline-controlled transactivator protein tTA-Advanced	Clontech (USA)
pBI-EGFP	allows expression of a gene of interest and marker gene of eGFP under control of the bi-directional tTA-responsive promotor	Clontech (USA)
**3Cpro gene source**		
pHAV3’^f^	bears a cDNA encoding a segment of human hepatitis A virus genome (strain HAS-15)	[138]/GenBank: X15463.1
**3Cpro-expressing plasmids**		
pBI-EGFP/3C	encodes 3Cpro and eGFP under control of bi-directed tTA-responsive promotor.	This study
pBI-EGFP/3CMut	encodes 3Cpro with Cys172→Ala mutation and eGFP under control of bi-directed tTA-responsive promotor.	This study

^a^Gift from Prof. Ari Helenius (Institute of Biochemistry, Swiss Federal Institute of Technology, Zurich, Switzerland).

^b^Gift from Dr. Richard Pagano (Mayo Clinic, Rochester, MN, USA).

^c^Gift from Dr. Tamotsu Yoshimori (National Institute for Basic Biology, Okazaki, Japan).

^d^Gift from Dr. Joel Swanson (University of Michigan Medical School, MI, USA).

^e^Gift from Dr. Felipe X. Pimentel-Muiños (Centro de Investigation del Cancer, Universidad de Salamanca-CSIC, Salamanca, Spain).

^f^Gift from Dr. Eugene Snezhkov (M.M. Shemyakin and Yu.A Ovchinnikov Institute of Bioorganic Chemistry RAS, Moscow, Russia).

^g^Plasmid was distributed through Addgene. Add #, Addgene plasmid number.

**Table 2:** **Primers used**

**Primer**	Sequence*
Bi3Cf	GCAGAA**GATATC** *GCCACCATG*TCAACTCTAGAAATAGCAGGA
Bi3Cr	CACTTT**GCTAGC**TTACTGACTTTCAATTTTCTTATC
Bi3Cm-f	GGTCTTCCCGGGATG***GCT***GGTGGGGCCCTAGTG
Bi3Cm-r	CACTAGGGCCCCACC***AGC***CATCCCGGGAAGACC
rt-3Cf	GGTTCAGTTTGGAGTTGGTGA
rt-3Cr	TTCCTCTCCATGCCTGATCT
rt-GAPDHf	GGTCGTATTGGGCGCCTGGTCACC
rt-GAPDHr	CACACCCATGACGAACATGGGGGC

*Restriction sites are boldfaced, Kozak sequence is italic, sequence corresponding to Cys172 → Ala mutation is boldfaced and italized. All primers were from Evrogen (Russia).

### Cell culture and transfection

Human lung carcinoma A549 (ATCC No. CCL-185) and human lung epidermoid carcinoma Calu-1 (ATCC No. HTB-54) cell lines were cultured in conventional media (DMEM/F-12, 10% FBS, 0.3 mg/ml glutamine) at 37°C in humidified atmosphere of 5% CO_2_.

A549 and Calu-1 Tet-Off Advanced monoclonal cell lines were established according to protocol of the supplier using the Tet-Off Advanced Inducible Gene Expression System (Clontech, USA) and were further maintained in 0.2 mg/ml G418.

Transfections were performed using Lipofectamine 2000. Briefly, cells were cultured as described above in POC-R chambers (PeCon GmbH, Germany), 24- or 96-well plates for 18–24 h until 80-90% confluence. Three hours before transfection, the media was replaced with fresh. Plasmid-Lipofectamin 2000 complexes were prepared following the protocol of the manufacturer in serum-free OptiMEM and added to cell cultures. When cotransfecting, pBI-EGFP and pBI-EGFP/3C were added to pER-RFP in a mass ratio of 1:1; to other plasmids, 10:1.

### Construction of 3Cpro-expressing vectors

The DNA fragment encoding 3Cpro was amplified by polymerase chain reaction (PCR) from pHAV-3′ plasmid using primers Bi3Cf and Bi3Cr; the amplified product was digested with EcoRV and NheI and cloned into pBI-EGFP digested with NheI and PvuII. The structure of the plasmid obtained (named pBI-EGFP/3C) was confirmed by sequencing.

The construction of a gene encoding catalytically inactive 3Cpro with Cys172 → Ala mutation was implemented in two steps by overlap extention PCR. At the first step, two overlapping fragments of 3Cpro gene with mutation were amplified by PCR from pBI-EGFP/3C using Bi3Cm-f/Bi3Cr and Bi3Cf/Bi3Cm-r pairs of primers. At the second step, the fragments obtained were used as primers and template in overlap extension PCR followed by the amplification of the full-length sequence with primers Bi3Cf/Bi3Cr. The resulting DNA product was digested with EcoRV and NheI and cloned into pBI-EGFP digested with NheI and PvuII. The structure of the plasmid obtained was confirmed by sequencing.

### Confocal microscopy

Confocal Microscopy was performed using a Carl Zeiss Axiovert 100 LSM510 META system with an Incubator XL-3 (PeCon GmbH, Germany) at 37°C. General cell morphology was evaluated in flat-bottom 24-well plates using an EC Plan-Neofluar 10×/0.30 M27 objective (Carl Zeiss, Germany). Subcellular structures were analyzed in POC-R Chambers and imaged using EC Plan-Neofluar 40×/1.30 Oil DIC M27 or EC Plan-ApoChromat 63×/0.75 Oil Korr objectives (Carl Zeiss, Germany).

The following excitation wavelength/emission filter settings were used for fluorescent proteins and dyes: 488 nm/510-530 nm for EGFP; 543 nm/615 nm long pass for RFP, mRFP, mKate2, propidium iodide, and MR-(RR) reagent (Magic Red Cathepsin B Detection Kit); 514 nm/515-570 nm for YFP and eYFP; 458 nm/470-500 nm for CFP; 405 nm/420-480 nm for Hoechst 33258; 405 nm/420-480 nm for ER-Tracker Blue-White DPX; 543 nm/580-680 nm for FLICA reagent (Caspase Detection Kit) and Cy3 (Annexin V-Cy3 Assay Kit); and 405 nm/510 nm long pass for Lucifer Yellow.

During time-lapse confocal microscopy experiments, cells were cultured in POC-R Chambers placed in a Heating Insert P (PeCon GmbH) at 37°C in humidified atmosphere of 5% CO_2_. The interval between scans was 5 minutes.

### Electron microscopy

48 h post-transfection (p.t.) with pBI-EGFP or pBI-EGFP/3C, cells were trypsinized, pelleted, washed with PBS, and resuspended in a fixative solution (0.2 M cacodylic acid-NaOH buffer, pH 7.5, and 2% glutaraldehyde). Sections were cut on an LKB III ultratome (Sweden) and examined under a JEM-100CX electron microscope (JEOL, Japan) at accelerating voltage of 80 kV.

### Analysis of 3Cpro cytotoxic effect

Cells were cultured in flat-bottom 96-well plates (Corning, USA) and transfected with pBI-EGFP and pBI-EGFP/3C plasmids. Every 24 h p.t., cells from 4 wells for each transfection variant were trypsinized and a percentage of eGFP-expressing cells was calculated using hemacytometer and microscope Olympus CKX-40 with excitation/emission filter set for green fluorescence. Growth media was replaced every 48 h.

### Characterization of dead cells

Chromatin condensation, integrity of plasma membrane, and maintenance of mitochondria potential were evaluated as follows: cells were stained with Hoechst 33258 (20 μg/ml, 20 min at 37°C), PI (10 μg/ml, 5 min at 37°C), and Rh123 (10 μg/ml, 15 min at 37°C), washed by cold PBS, placed in fresh media, and examined under a confocal microscope. Phosphatidylserine externalization was detected with an AnnexinV-Cy3 Apoptosis Detection Kit, and activation of caspases was assayed using an Image-iT LIVE Red Poly Caspases Detection Kit following the suppliers’ protocol.

### Counting cells with different morphology

Cells with different morphology (designated as normal, vacuolated, and rounded/blebbed) were counted every 24 h p.t. Cells with three or more cytoplasmic vacuoles occupying more than 20% of visible cell area were considered vacuolated. Cells demostrating rounded shape, shrinkage and smooth or blebbed plasma membrane were considered rounded/blebbed. Images of each culture with at least 200 cells were examined. Data from two independent experiments were averaged.

### Treatment with inhibitors of enzymes and cell processes

All substances were added with fresh conventional growth media (18–20 h p.t. immediately after Lipofectamin-DNA complexes were removed from cells) and incubated for 24 or 48 h. Concentrations of substances in the media were 10–100 μM for Z-VAD-fmk, 10–100 μM for z-FA-fmk, 50 μM for necrostatin-1, 1 nM for bafilomycin A1, 10 mM for 3-methyladenine, 30 μM for PD98059, 36 nM for Sc-353669, 60 μM for colchicine, 1.8 μM for doxorubicin and 1.5 μM for filipin. In the case of substances dissolved in DMSO, the same amounts of the solvent were added to media of control cell cultures to exclude solvent-induced effects. Functionality of Z-VAD-fmk, z-FA-fmk, 3-methyladenine and necrostatin-1 was confirmed in model experiments described in Additional file 2: Figure S1. Molecular masses of the inhibitors were verified by mass spectrometry analysis (data not shown).

### Treatment with Lucifer Yellow and Neutral Red

Lucifer Yellow (LY) and Neutral Red (NR) were added to cell cultures with fresh conventional growth media 48 or 72 h p.t. The concentrations of substances in the media were 1 mM for LY and 2 mM for NR. Cells were incubated in LY solution for 2, 4, or 12 h and in NR solution for 10 min. After the incubations, the cells were rinsed with cold PBS three times, placed in fresh media, and examined microscopically.

### Reverse transcription PCR analysis

Cells were grown in 25 cm^2^ plates, transfected with pBI-EGFP, pBI-EGFP/3C, or pBI-EGFP/3CMut and collected 48 h p.t. RNA from the cells was isolated using the RNAqueous kit (Life Technologies, USA) according to the suppliers’ protocol. The RNA samples obtained were treated with 1–5 units of RQ1 DNAse (37°C, 1 h; inactivation, 65°C, 30 min). Reverse transcription was carried out with the High Capacity cDNA Reverse Transcription Kit (Applied Biosystems, USA). The obtained cDNA samples were subjected to PCR with rt-GAPDHf/rt-GAPDHr and rt-3Cf/rt-3Cr pairs of primers to amplify DNA fragments encoding GAPDH and 3Cpro, respectively, according to the following program: 94°C 4 min; 26 cycles: 94°C 30 s, 60°C 30 s, 72°С 1 min; and 72°C 15 min.

## Additional files

Additional file 1:
**Supplemental video.** Time-lapse confocal microscopy of a vacuolated A549/3Cpro cell in the period of 50–60 h p.t. Additional file 2: Figure S1. Functional tests of inhibitors. **Figure S2.** Effect of inhibitors on 3Cpro-induced vacuolization. **Figure S3.** Effect of inhibitors on cell viability.
