# Supplementary material for: Protease 3C of hepatitis A virus induces vacuolization of lysosomal/endosomal organelles and caspase-independent cell death
Source: BMC Cell Biol. 2015 Feb 27;16:4. doi: 10.1186/s12860-015-0050-z (PMC4355371; doi:10.1186/s12860-015-0050-z)
Supplement: Additional file 2: Figure S1. — Functional tests of inhibitors. Figure S2. Effect of inhibitors on 3Cpro-induced vacuolization. Figure S3. Effect of inhibitors on cell viability. [file 12860_2015_50_MOESM2_ESM.pdf]

Supplemental Figures

Figure S1

A

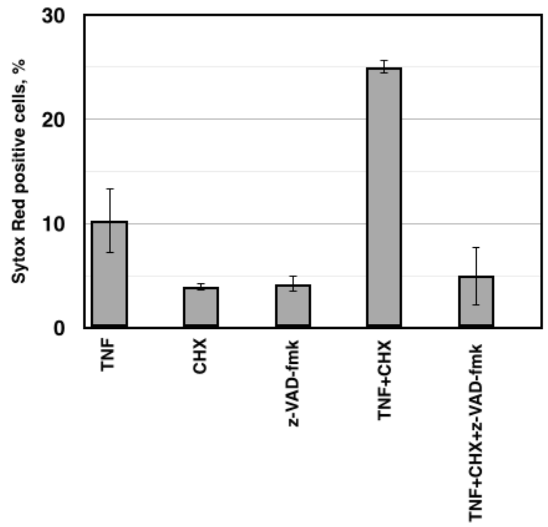

B

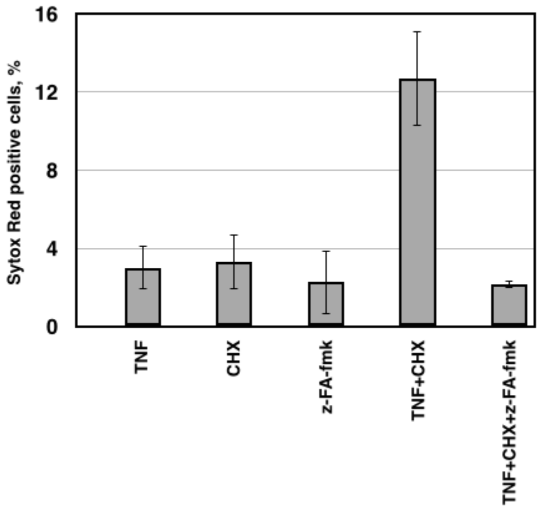

C

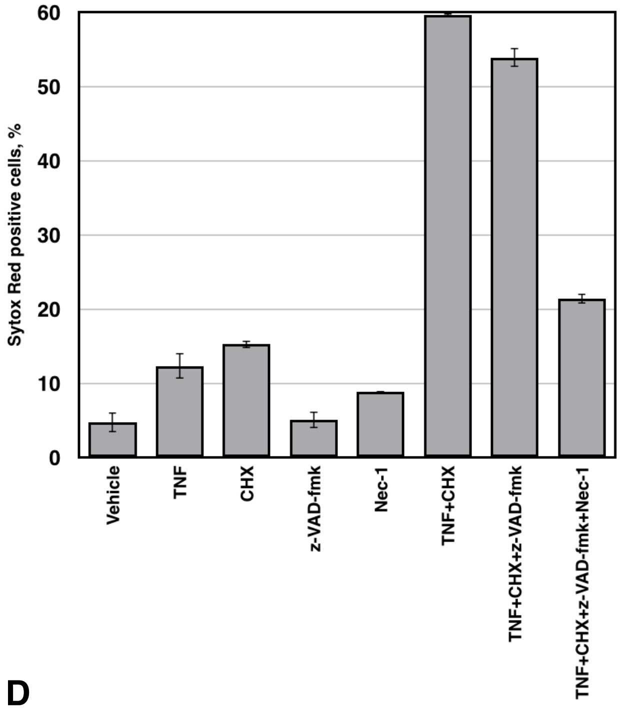

D

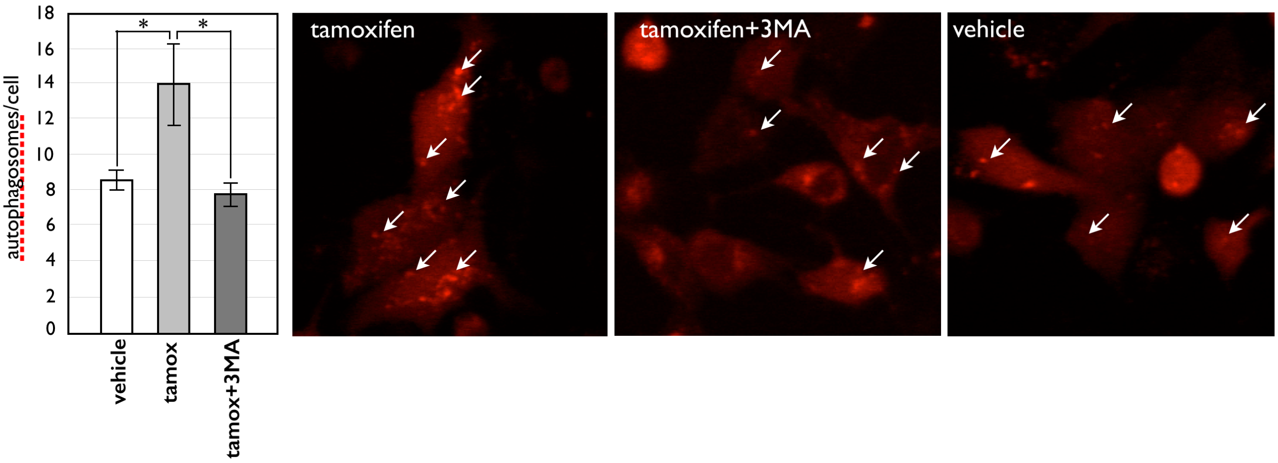

Figure S1 – Functional tests of inhibitors. A, B. Tests of z-VAD-fmk and z-FA-fmk. To induce apoptosis, approx. 50,000 A549 cells were seeded in triplicates in 96-well plate and treated with 50 ng/ml TNF-alpha (Meridian Life Sci, USA) and 2 μg/ml cycloheximid (CHX) (Sigma, USA). To prevent apoptosis, 50 μM z-VAD-fmk or 50 μM z-FA-fmk were added. Cells were incubated for 18 h (for z-FA-fmk) or 24 h (for z-VAD-fmk). After the incubation, cells were stained with Sytox Red Dead Cell Stain (LifeTechnologies, USA) and analyzed on BD Accuri C6 flow cytometer. Bars on the graph show averaged number of Sytox Red-positive cells; ticks show standart deviation. **C.** Test of necrostatin-1. To induce RIP-1 dependent necroptosis, approx. 50,000 Jurkat cells were seeded in duplicates in 96-well plate and treated with 100 ng/ml TNF, 50 μM z-VAD-fmk and 2 μg/ml cycloheximid (CHX) (as described in Sosna et al., Cell. Mol. Life. Sci., 2014, 71). To inhibit necroptosis, 50 μM necrostatin-1 (Nec-1) was added. After the incubation, cells were stained with Sytox Red Dead Cell Stain and analyzed on BD Accuri C6 flow cytometer. Bars on the graph show averaged number of Sytox Red-positive cells; ticks show standart deviation. **D.** Test of 3-methyladenine. Approximately 50,000 A549 cells were seeded in triplicates in 48-well plate, transiently transfected with pmRFP-LC3 (as described in the Materials and Methods) and treated with tamoxifen (tamox) alone (according to the manufacturer' protocol, Cayman Chemical, USA) or with addition of 10 mM 3-methyladenine (3MA) for 48 h. Equal amount of vehicle (DMSO) was added to control cells. Mean numbers of autophagosomes per cell was counted for 20 cells for each well. Bars on the graph show averages of the mean values for the each treatment group; ticks show standard deviation. Significance of difference between groups was calculated by Kruskal-Wallis sum rank test. Stars show statistical significance with $p\text{-value}<0.05$.

Figure S2

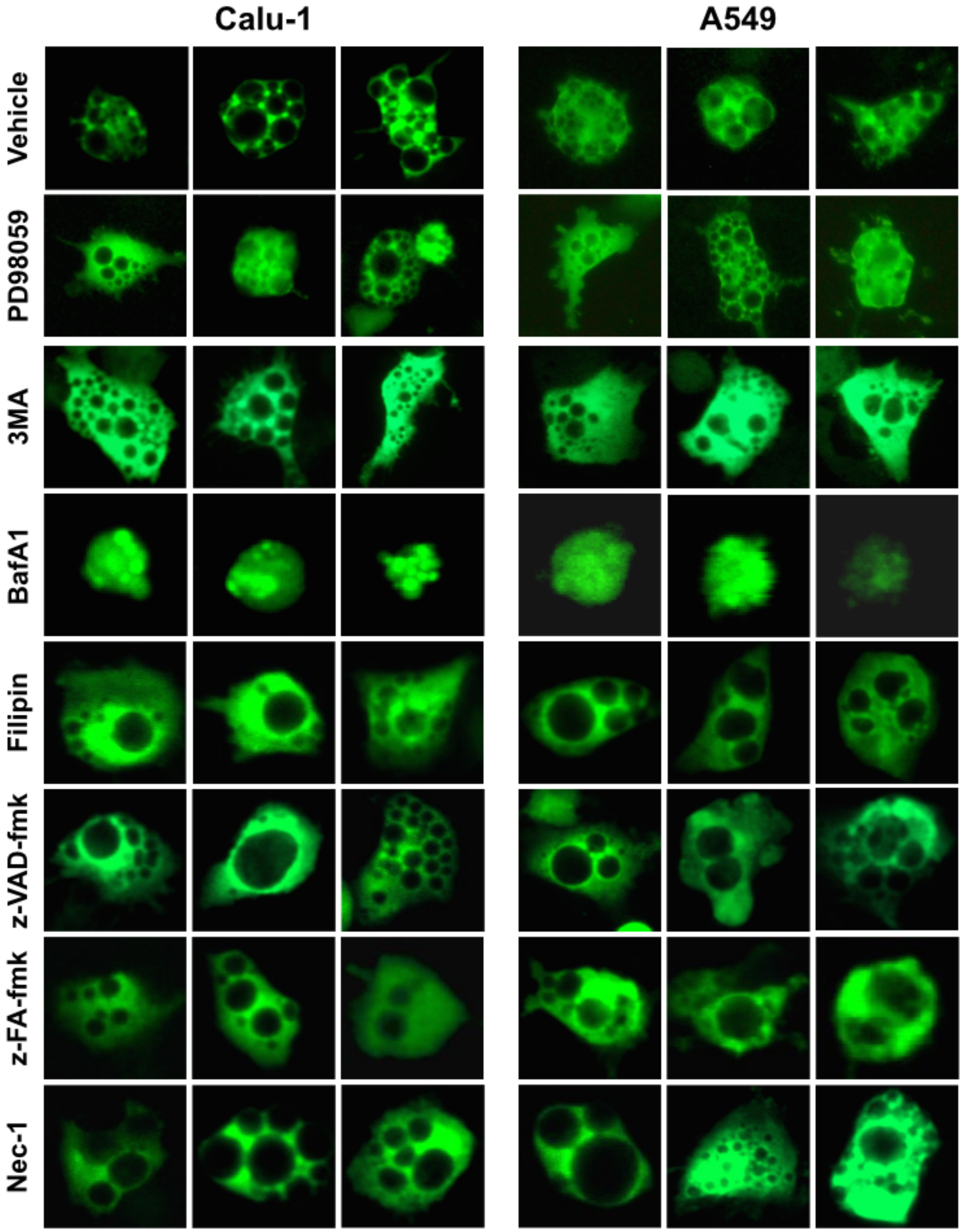

Figure S2 – Effect of inhibitors on 3Cpro-induced vacuolization. Images of A549/3C and Calu-1/3C cells treated with inhibitors (as described in the Materials and Methods), 48 and 72 h p.t. Amongst the all inhibitors used, only Bafilomycin A1 (BafA1) prevented vacuolization.

Figure S3

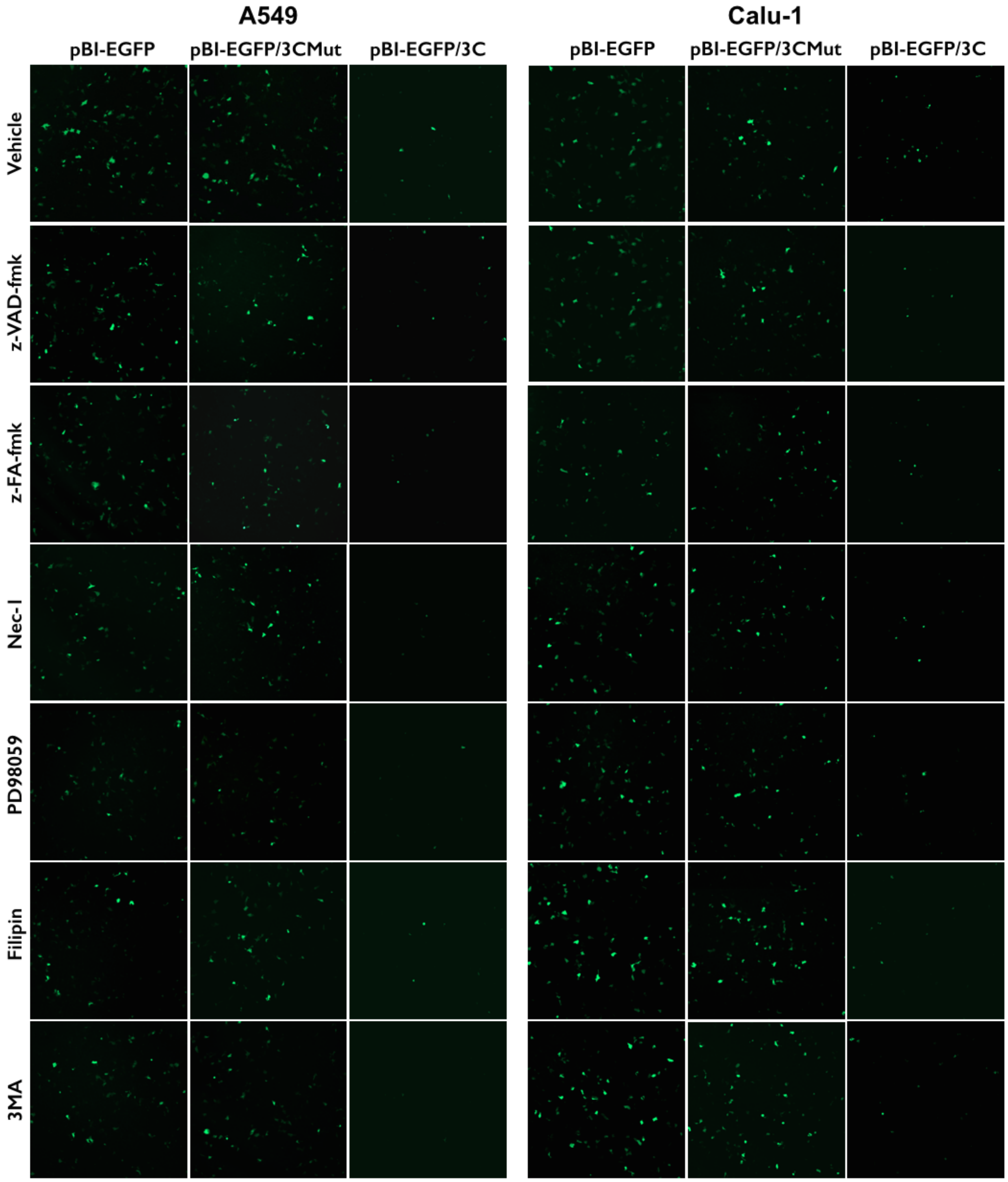

Figure S3 – Effect of inhibitors on cell viability. Images of A549 and Calu-1 cultures transfected with pBI-EGFP, pBI-EGFP/3C or pBI-EGFP/3CMut and treated with ihnibitors (as described in the Materials and Methods), 72 h p.t.
